# High prevalence and diversity of species D adenoviruses (HAdV-D) in human populations of four Sub-Saharan countries

**DOI:** 10.1186/1743-422X-11-25

**Published:** 2014-02-11

**Authors:** Maude Pauly, Eileen Hoppe, Lawrence Mugisha, Klara Petrzelkova, Chantal Akoua-Koffi, Emmanuel Couacy-Hymann, Augustin Etile Anoh, Arsène Mossoun, Grit Schubert, Lidewij Wiersma, Sabwe Pascale, Jean-Jacques Muyembe, Stomy Karhemere, Sabrina Weiss, Siv Aina Leendertz, Sébastien Calvignac-Spencer, Fabian H Leendertz, Bernhard Ehlers

**Affiliations:** 1 Project group “Epidemiology of Highly Pathogenic Microorganisms”, Robert Koch Institute, Berlin 13353, Germany; 2 Division 12 “Measles, Mumps, Rubella and Viruses affecting immunocompromised patients”, Robert Koch Institute, Berlin 13353, Germany; 3 Department of Wildlife and Animal Resource Management, Makerere University, Kampala 7062, Uganda; 4 Conservation & Ecosystem Health Alliance (CEHA), Kampala 34153, Uganda; 5 Institute of Vertebrate Biology, Academy of Sciences, Brno 60365, Czech Republic; 6 Department of Pathology and Parasitology, University of Veterinary and Pharmaceutical Sciences, Brno 61242, Czech Republic; 7 Centre de Recherche pour le Developpement, University Alassane Ouattara of Bouake, Bouake 01 BP V18, Côte d’Ivoire; 8 LANADA/Laboratoire Central de Pathologie Animale, Bingerville 206, Côte d’Ivoire; 9 Institut National de Recherche Biomédicale, Kinshasa 1197, Democratic Republic of Congo; 10 ViroscienceLab, Erasmus Medical Centre, Rotterdam 3015, The Netherlands

**Keywords:** Adenoviridae, Human adenovirus D, Genotype, Sub-Saharan Africa, PCR

## Abstract

**Background:**

Human adenoviruses of species D (HAdV-D) can be associated with acute respiratory illness, epidemic keratoconjunctivitis, and gastroenteritis, but subclinical HAdV-D infections with prolonged shedding have also been observed, particularly in immunocompromised hosts. To expand knowledge on HAdV-D in Sub-Saharan Africa, we investigated the prevalence, epidemiology and pathogenic potential of HAdV-D in humans from rural areas of 4 Sub-Saharan countries, Côte d’Ivoire (CI), Democratic Republic of the Congo (DRC), Central African Republic (CAR) and Uganda (UG).

**Methods:**

Stool samples were collected from 287 people living in rural regions in CI, DRC, CAR and UG. HAdV-D prevalence and diversity were determined by PCR and sequencing. A gene block, spanning the genes pV to hexon, was used for analysis of genetic distance. Correlation between adenovirus infection and disease symptoms, prevalence differences, and the effect of age and gender on infection status were analyzed with cross tables and logistic regression models.

**Results:**

The prevalence of HAdV-D in the investigated sites was estimated to be 66% in CI, 48% in DRC, 28% in CAR (adults only) and 65% in UG (adults only). Younger individuals were more frequently infected than adults; there was no difference in HAdV-D occurrence between genders. No correlation could be found between HAdV-D infection and clinical symptoms. Highly diverse HAdV-D sequences were identified, among which a number are likely to stand for novel types.

**Conclusions:**

HAdV-D was detected with a high prevalence in study populations of 4 Sub-Saharan countries. The genetic diversity of the virus was high and further investigations are needed to pinpoint pathological potential of each of the viruses. High diversity may also favor the emergence of recombinants with altered tropism and pathogenic properties.

## Background

Adenoviruses are non-enveloped icosahedral viruses with a linear, double-stranded DNA genome that can cause a wide spectrum of clinical diseases, including acute respiratory illness, epidemic keratoconjunctivitis, acute hemorrhagic cystitis, hepatitis, myocarditis and gastroenteritis [1,2]. Primary infection with human adenoviruses (HAdV) or reactivation of persistent HAdV can lead to severe systemic diseases in immunocompromised hosts, such as bone marrow and solid organ transplant recipients and patients with AIDS [3-5]. Based on serology, whole-genome sequencing, and phylogenomics, eight HAdV species (HAdV-A to -G) are differentiated within the genus *Mastadenovirus,* comprising a total of 69 recognized HAdV types. Most HAdV types belong to species D and the majority of these HAdV-D types have been detected in HIV-positive patients [6-8]. Knowledge about the pathogenicity of HAdV-D types is limited. Types D8, D19, D37 and D54 are known to cause epidemic keratoconjunctivitis [9-13]. The virulent type D53, a recombinant between D8, D22, D37 and at least one unknown HAdV-D type, showed a modified tropism and induced inflammation of the cornea [14]. Another recombinant type, D56, was involved in fatal pneumonia in a neonate and keratoconjunctivitis in three adults [15]. Type D36 has been associated with obesity in animals and humans [16] and types D65 and D67 were detected in the stool of children with gastroenteritis [8,17]. The pathogenicity of the HAdV-D types frequently shed by patients with AIDS remains controversial [3,18-20]. Prolonged fecal and urinary shedding of different HAdV-D types and of recombinants has been observed [18,21]. Hence it has been proposed that recombination might play a greater role in HAdV-D evolution than base substitution [6]. Since HIV/AIDS as well as gastroenteritis are common diseases in Sub-Saharan Africa and PCR-based and sequence-confirmed studies on the prevalence of HAdV-D in Sub-Saharan Africa are scarce [22,23], we analyzed the occurrence of HAdV-D in people from four Sub-Saharan countries; the Côte d’Ivoire (CI), the Democratic Republic of the Congo (DRC), the Central African Republic (CAR), and Uganda (UG). We assessed possible connections between HAdV-D shedding and clinical symptoms or demographic data, and in addition, we partially characterized the molecular isolates by comparison of minimum genetic distances within the pV-hexon gene block.

## Results

HAdV-D generic nested PCR and sequencing revealed a HAdV-D prevalence of 66% (95% CI 56-76%) in CI, 48% (95% CI 38-58%) in DRC, 28% (95% CI 13-51%) in CAR (adults only), and 65% (95% CI 53-75%) in UG (adults only). The prevalence in CI was significantly higher than in DRC (P < 0.01). When comparing the adult populations in all four countries, the prevalence in CI and UG was significantly higher than in DRC and CAR (p < 0.05). In CI and DRC 100% and 68% of the younger children and 71% and 50% of the older children and adolescents were HAdV-D positive (Table 1) and the proportion of infected people decreased further to 64% and 39% in the adults in CI and DRC, respectively. Overall there was a significant decrease in the proportion of infected individuals with increasing age group (regression coefficient -0.6, p < 0.05). Gender had no significant effect on infection status (p > 0.100). The prevalence per village ranged from 45-100% (data for individual villages not shown) and there was overall no significant difference between the villages (p > 0.05) (mean n/village = 16.7, range 2-38). The logistic regression model explained 11% of the variance in the dataset (pseudo R-square = 0.1064).

**Table 1:** Human adenovirus species D (HAdV-D) detection and clinical symptoms

	**Number of analysed individuals**	**% analysed individuals**	**Number of HAdV-D positive individuals**	**% HAdV-D positive individuals**
**Côte d’Ivoire**				
Study group	95	100	63	66
0-5 years	4	4	4	100
6-19 years	7	7	5	71
20-77 years	84	88	54	64
Male	42	44	30	71
Female	53	56	34	63
Asymptomatic	45	47	15	33
Symptomatic	50	53	35	70
Abdominal pain	21	22	14	67
Diarrhea	3	3	3	100
Nausea	1	1	1	100
Ocular disease	6	6	3	50
Fever	10	11	9	90
Respiratory disease	8	8	6	75
Headache	19	20	12	63
**Democratic Republic of the Congo**				
Study group	105	100	50	48
0-5 years	19	18	13	68
6-19 years	30	29	15	50
20-78 years	56	53	22	39
Male	51	49	27	53
Female	54	51	23	43
Asymptomatic	41	39	36	88
Symptomatic	64	61	27	42
Abdominal pain	46	44	18	39
Diarrhea	4	4	0	0
Nausea	1	1	0	0
Ocular disease	0	0	0	0
Fever	9	9	3	33
Respiratory disease	18	17	8	44
Headache	14	13	6	43

To investigate the pathogenicity of HAdV-D, we tested for a correlation between HAdV-D shedding and clinical symptoms. Overall, 57% of the study participants reported at least one clinical symptom. There was no correlation between infection status and individual clinical signs, or between infection status and poor health status, i.e. individuals that reported symptoms (p > 0.05) (Table 1).

**Figure 1:**
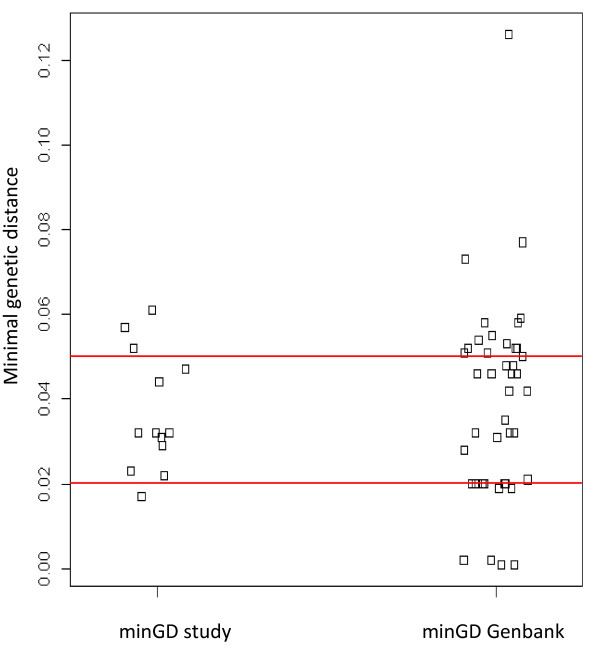
**Comparison of observed minimum genetic distances.** In this strip-chart, observed minimum genetic distances (minGD) of human adenovirus species D (HAdV-D) types are plotted. Left panel: minGD was determined for the 13 HAdV-D sequences of this study in relation to 43 HAdV-D types from Genbank. Right panel: minGD was determined for every HAdV-D type from Genbank in relation to the other 42 Genbank types. The red lines indicate the first quartile (25%) at 0.02 and the third quartile (75%) at 0.05 of the minGD values from the 43 Genbank types. The interquartile range (0.05-0.02) gives the range of the middle 50% of the observed minGD values of the Genbank types.

We finally analyzed if the sequences of the current study were derived from novel HAdV-D types. For this purpose, we attempted to amplify a 4.8 kb fragment spanning the genes pV-hexon gene block from 35 randomly chosen, HAdV-D positive samples from CI, DRC, CAR and UG. Fragments were obtained from 14 samples after 2^nd^ or 3^rd^ round of Long-Distance PCR and completely sequenced. Thirteen different HAdV-D sequences were obtained. Since in 2 samples from UG close to identical sequences were identified, only one of them (Hu4555_UG) was included in further analysis. BLAST analysis of the hyper-variable Loop 2 region from the 13 sequences revealed a pairwise identity of 86.4%-100% to known HAdV-D types (HAdV-D types 9, 13, 15, 17, 23, 25, 27, 29, 30, 32 47, 48, 56, 65, 67). We then compared the minimum genetic distances (minGD) of the pV-hexon sequences from 43 known HAdV-D types with those of the 13 types identified in this study. First, we determined minGD between the 43 recognized types (see *Methods*) to estimate the range of intertypic minGD. 50% of all values lay between 0.02 and 0.05 nucleotide substitution per site (Figure 1). Then, we determined minGD between the 13 sequences generated in this study and any recognized type: here 84.6% of all values lay between 0.02 and 0.05 nucleotide substitutions per site (Figure 1; Table 2). All 13 unique sequences identified in this study exhibited minGD >0.02; 5 sequences even exhibited a minGD >0.04, which outperformed >50% of intertypic minGD (Figure 1; Table 2). Comparable results were obtained by using estimated instead of observed minGD (Table 2) and by analyzing only the region containing the hyper-variable hexon loops (Loops 1 and 2). These loops represent the major target for antibodies, are involved in immune escape, are particularly prone to recombination events and are among the target sequences for the characterization of HAdV types. Although more sequence information would be desirable, our data already point at 5 of 13 unique sequences likely representing novel types (under the conservative assumption that intertypic minGD > intratypic minGD in at least 50% cases). Recombination analysis of our data confirmed the tendency of HAdV-D to recombine in the hyper-variable loop region of the hexon gene (data not shown).

**Table 2:** Comparison of the minimum genetic distance values of HAdV-D types

**Sample**	**Observed genetic distances**						**Estimated genetic distances**					
	**pV-hexon gene**			**Hypervariable loops**			**pV-hexon gene**			**Hypervariable loops**		
	**minGD study**	**n minGD Genbank**	**% minGD Genbank**	**minGD study**	**n minGD Genbank**	**% minGD Genbank**	**minGD study**	**n minGD Genbank**	**% minGD Genbank**	**minGD study**	**n minGD Genbank**	**% minGD Genbank**
		**<**	**<**		**<**	**<**		**<**	**<**		**<**	**<**
		**minGD study**	**minGD study**		**minGD study**	**minGD study**		**minGD study**	**minGD study**		**minGD study**	**minGD study**
**Hu4751_CI**	0.02	4	9.30	0.00	6	13.95	0.02	4	9.30	0.00	0	0.00
**Hu4806_CI**	0.02	13	30.23	0.03	11	25.58	0.02	10	23.26	0.02	11	25.58
**Hu4719_CI**	0.02	13	30.23	0.04	13	30.23	0.02	10	23.26	0.04	11	25.58
**Hu4787_CI**	0.03	15	34.88	0.06	18	41.86	0.03	13	30.23	0.07	13	30.23
**Hu4152_DRC**	0.03	16	37.21	0.04	13	30.23	0.03	13	30.23	0.06	13	30.23
**Hu4813_CI**	0.03	16	37.21	0.02	10	23.26	0.03	13	30.23	0.01	11	25.58
**Hu4882_CI**	0.03	16	37.21	0.07	18	41.86	0.04	17	39.53	0.11	19	44.19
**Hu4746_CI**	0.03	16	37.21	0.07	18	41.86	0.03	16	37.21	0.25	22	51.16
**Hu4109_DRC**	0.04	22	51.16	0.12	20	46.51	0.05	22	51.16	0.26	22	51.16
**Hu4108_DRC**	0.05	26	60.47	0.11	20	46.51	0.05	22	51.16	0.13	20	46.51
**Hu4564_UG**	0.05	31	72.09	0.12	20	46.51	0.06	29	67.44	0.29	22	51.16
**Hu4214_DRC**	0.06	37	86.05	0.17	33	76.74	0.07	34	79.07	0.67	35	81.40
**Hu4555_UG**	0.06	37	86.05	0.16	26	60.47	0.07	37	86.05	0.50	31	72.09

minGD study: minimum genetic distance determined for the 13 HAdV-D sequences of this study in relation to 43 HAdV-D types from Genbank; minGD Genbank: minimum genetic distance determined for every HAdV-D type from Genbank in relation to the other 42Genbank types; CI: Côte d`Ivoire; DRC: Democratic Republic of the Congo; UG: Uganda.

## Discussion

In this study, we estimated a relatively high prevalence of HAdV-D in several rural study populations in Sub-Saharan countries. The overall prevalence in CI was significantly higher than in DRC, which was also reflected by the fact that the proportion of HAdV-D individuals across all age classes was higher in CI compared to DRC (Table 2). UG also had a particularly high prevalence when comparing the adult population only. Since we were able to explain only 11% of the variance in the dataset with the model, there are likely to be many factors that influence the difference in HAdV-D occurrence which have not been included in this study. Considering the fecal-oral nature of HAdV transmission, such factors may be sources of local water supply and hygiene measures including toilet facilities. Also, nutrition and the local occurrence of HIV and other infections might play a role in the susceptibility to, and shedding of, HAdV-D. HIV prevalence was not determined for the populations in the present study, but it is possible to speculate that the high prevalence in CI is partly a result of the relatively high HIV-1 prevalence in the Tai area (7.2%) [24].

Various sampling strategies and detection methods limited the feasibility of direct comparison of our cross-sectional study to other studies (Additional file 1: Table S1) [22,23,25-35]. Study participants were within limited age groups, showed specific symptoms and/or different life styles. Contrary to other studies, we applied a generic nested PCR using non-degenerate primers that target the hexon gene of all known HAdV-D types and sequenced all positive samples. Most primers implemented in other studies were degenerate and targeted several HAdV species and if HAdV were confirmed by sequencing, this was only performed for a selection of samples (Additional file 1: Table S1). This might have resulted in a considerable underestimation of the HAdV-D prevalence.

Our study included participants of all age groups ranging from young children, older children/adolescents to adults. We were able to detect that younger individuals shed HADV-D significantly more frequently, which shows on one hand that exposure to this infection likely occurs early in life and on the other hand that adults might develop immunity leading to a reduction in HAdV-D shedding. It is not clear, whether this high prevalence can be explained by a general high sensitivity of children to any infection, or by a more likely ingestion of contaminated material in young children compared to adults. Although mothers should in theory be at higher risk of getting infection through baby care, no difference between men and women regarding HAdV-D shedding was observed. This could indicate that the majority of infections occur via generally available sources in the villages.

It has been shown that some HAdV-D types induce specific symptoms [8,9,14,16,17]. In our study, HAdV-D sequences could not be finally assigned to specific HAdV-D types, which makes it less likely to find a correlation between HAdV-D infection and an individual symptom. In addition, the symptoms induced by HAdV-D types are not pathognomonic and can be associated with different pathogens. To determine the effect of specific HAdV-D types, it would be necessary, to exclude other pathogens causing similar symptoms and to perform type-specific laboratory analyses.

We analyzed genetic distances to further characterize the HAdV-D types involved in this study. Historically, novel AdV types have been determined using serological assays based on recognition of specific epitopes on the viral capsid and on biological properties (oncogenic, haemagglutinating and morphological properties). Nowadays, phylogenetic analyses of complete sequences of the capsid proteins, hexon, fiber and penton base, have been shown to be good predictors for new types and for detection of recombination events [36-38]. We analyzed a 4.8 kb long sequence comprising the pV-hexon gene block. Molecular divergence within the hexon protein, the most significant protein for classification and recognition of types, can be used to estimate whether sequences are likely to represent novel AdV types [39,40]. Sequencing of the hyper-variable Loop 2 region of the hexon gene has been proposed to be sufficient for HAdV-typing [39]. For HAdV-D, a genetic divergence ranging from 0.3 to 2,7% has been reported [40]. Although analysis of the pV-hexon sequence alone does not permit definite typing of HAdV-D, since recombination may have occurred in other genes, comparison of minimum genetic distances between recognized types and the study sequences strongly suggested that novel HAdV-D types are involved in our study (Figure 1; Table 2).

## Conclusions

This study shows that HAdV-D is prevalent in rural populations across Sub-Saharan Africa, and that the virus is more frequently shed by younger than older individuals. HAdV-D shedding could not be linked to disease symptoms, but specific types were not investigated. The diversity of the virus was high, which may include pathogenic variants and favor the emergence of recombinants with altered tropism and pathogenic properties.

## Methods

### Subjects, sampling and statistical analysis

Between 2011 and 2012, we collected stool samples from individuals who have lived in or next to the tropical rain forest in Western (Taï National Park in CI) and Central (Salonga National Park in DRC) Africa for several years. The study populations were predominantly hunters, livestock breeders and cultivators with (CI) or without (DRC) regular contact to other populations. Volunteer participants (93 male; 107 female) were recruited from 12 villages (CI, *n* = 8; DRC, *n* =4). Participants were between 0 and 77 years old (mean = 41) in CI, and between 0 and 78 years old (mean = 28) in DRC. A basic clinical examination was performed by a trained medical professional and, when necessary, free treatment and medical advice were provided. If treatment was not possible on site, individuals were referred to an appropriate medical facility. The final written medical history included age, sex, location of residence, and information about current medical condition. Participants were assigned to one of three age groups: young children (0-5 years old), older children and adolescents (6-19 years old), and adults (20 year and older). The same sampling procedure was applied in CAR and UG, where stool samples were collected from adult male field assistants of Dzanga-Sangha Protected Areas (CAR, *n* = 18) and Bwindi National Park (Uganda, *n* = 69). Clinical data were not recorded for these 2 groups. Before sampling, the aim of the study, as well as the possibility to quit the study at any point, was explained individually in the local language. An individual study number was assigned to every participant in order to protect the privacy of the participant. Written informed consent was obtained from every study participant before sampling and the collection was approved by the responsible ethic commission of every country and was performed according to the declaration of Helsinki. The samples were conserved in liquid nitrogen and later stored at -80°C. The export to Germany occurred on dry ice with the appropriate permissions. DNA extraction was performed using the Qiagen and Roboklon stool kits, according to the manufacturer’s instructions.

Descriptive statistics, prevalence estimation and the effects of demographic data were generated and analyzed in Stata v12.0, using cross tables with Fischer exact tests and logistic regression models in Stata v12.0 with HAdV-D status as dependent binomial factor and country, gender, age group and village as independent factors. Results from CAR and UG were used for prevalence estimation only.

### Specific nested HAdV-D PCR

For the generic detection of members of the species HAdV-D, non-degenerate primers were designed based on an alignment of published HAdV-D sequences (Table 3). These primers amplify a fragment of 354 bp in the first round, and of 322 bp in the second round of PCR. The target sequence lays at the end of the hexon gene (position 20 194 - 20 547 in the genome of HAdV-D36; Genbank accession number: GQ384080).The first round of the nested PCR was performed with 5 μl of DNA extracted from human feces as template, 12.7 μl of PCR mix (containing 1× AmpliTaq™ buffer, 2,0 mM MgCl2, 5% DMSO, dNTPs at 200 mM each, 2.0 units of AmpliTaq Gold Polymerase (Applied Biosystems) and 1 μM of each first round sense and antisense primer) and PCR-grade H_2_O ad 25 μl. In second round amplification, 1.5 μl of the first-round amplification product was added to 12.7 μl of PCR mix with 1 μM of each second round primers and H_2_O ad 25 μl. Thermocyclers of type TgradientS (Biometra, Germany) were used under the following conditions: activation of the polymerase at 95°C for 12 min and 45 cycles of denaturation (95°C, 30 s), annealing (63°C, 30 s), and elongation (72°C, 2 min), final elongation at 72°C for 10 min.

**Table 3:** Primers for specific and long-distance PCR

**PCR**	**Gene**	**HAdV species**	**1**^**st**^**-round primer (5′-3′)**		**2**^**nd**^**-round primer (5′-3′)**		**3**^**rd**^**-round primer (5′-3′)**		**Annealing temp. (C°)**	**Fragment size**
			**Sense**	**Anti-sense**	**Sense**	**Anti-sense**	**Sense**	**Anti-sense**		
Generic PCR	Hexon	HAdV-D	AAGGCCGTCA	GTGCGGCTGG	ACAACTCGGGCTTCACCGGC	GGCTGGTGCA	None	None	63	322 bp
			CCCTGCCCTT	TGCACTCTGA		CTCTGACCACG				
Long distance PCR	PVII-Hexon	HAdV-D	CGCCCAGCAA	GTGCGGCTGG	GGCAGGACTCGCAGACGAGC	GGTGGGCTCA	GCGCGGAAAC	GGGATGCGCC	60	4.8 kb
			TAACACCGGC	TGCACTCTGA		TCCATGGGGT	GTGTACTGGGT	ACATGACCCT		

All PCR products of expected size were purified and sequenced with the Big Dye terminator cycle sequencing kit (Applied Biosystems, Warrington, UK) on a 377 automated DNA sequencer (Applied Biosystems).

### Specific long-distance-PCR

Long-Distance nested PCR was performed using the TaKaRa-EX PCR system according to the instructions of the manufacturer (Takara Bio Inc., Otsu, Japan) and resulted in a fragment of 4.8 kb. The sense primers bind in the 3′-end of the pVII gene and the antisense primers in the 3′-end of the hexon gene (Table 3). The target sequence lays between positions 15 091 and 20547 of the genome of HAdV-D36. Depending on the DNA concentration, up to 7 μl of DNA, were added in the first round to 15.5 μl of PCR mix (containing 10× ExTaq buffer with MgCl2, dNTPs at 2.5 mM each, 5.0 units of Ex Taq Polymerase and 10 μM of each first round sense and antisense primer) and PCR-grade H_2_O ad 50 μl. In second and third round amplification, 1 μl of the reaction product generated in the preceding PCR round, was added to 15.5 μl of PCR mix with 10 μM of the respective primers and H_2_O ad 50 μl. Thermocyclers of type TgradientS (Biometra, Germany) were used under the following conditions for every round: activation of the polymerase at 94°C for 5 min and 15 cycles of, followed by 15 cycles of denaturation (98°C, 20 s), annealing (60°C, 30 s), and elongation (68°C, 8 min + 5 s) and a final elongation at 72°C for 30 min. All PCR products of expected size were purified and sequenced as described above.

### Sequence analysis

The 4.8 kb sequences determined in this study (n = 13) were added to a data set consisting of sequences of the pV-hexon gene block available in Genbank from completely sequenced HAdV-D genomes (n = 43) (listed in *Methods*). They were aligned with the ClustalW multiple alignment method (EMBL, Heidelberg, Germany) (Alignment 1). Another alignment was performed comprising only the 43 Genbank sequences (Alignment 2). In addition, the hyper-variable loop regions of the hexon gene were extracted from alignments, resulting in the alignments 1a and 2a. Conserved blocks were selected from these alignments, using Gblocks [41] as implemented in SeaView v4 [42]. This resulted in alignments of 4.556 (Alignment 1 and 2) and 1.065 nucleotides (Alignment 1a and 2a). Observed genetic distance matrices were obtained for every alignment with the program Geneious v6.1.6 [43]. Evolutionary distances were estimated using PAUP* v4.0 [44], based on the best fitting nucleotide substitution model (GTR + I + G), as determined using jModeltest v2.1.3 [45].

In the observed and estimated genetic distance matrices of the alignments 1 and 1a, the minimum genetic distance (minGD) was determined for every sequence of this study (minGD study 1-13) in relation to the 43 HAdV-D types from Genbank. Correspondingly, in the observed and estimated genetic distance matrix of alignment 2 and 2a the minGD was defined for every HAdV-D type from Genbank (minGD Genbank 1-43) in relation to the other 42 types. Subsequently, we visualized the 13minGD study values and the 43 minGD Genbank values in a strip chart (carried out with R software) (Figure 1) and assessed for every minGD study value the number and percentage of inferior minGD Genbank values (Table 2).

### Recombination analysis

With default settings in the Recombination Detection Program v.4.16 (RDP4), recombination events, likely parental isolates of recombinants and recombination break points were analyzed using the RDP, GENECONV, Chimaera, MaxChi and BOOTSCAN methods implemented in the RDP4 program [46,47].

### Genbank accession numbers of published adenovirus sequences used for comparison of genetic distances

Genbank accession numbers AB448769; AJ854486; JN226746; JN226747; AB562586; AF108105; AB448774; JN226749; FJ404771; JN226750; JN226751; JN226752;EF153474; JN226753; FJ824826; AB562587; JN226755; JN226756; JN226758; GQ384080; DQ393829; AB448778; JN226759;JN226760; JN226761; JN226762; JN226763; JN226764; AY875648; JN226757; EF153473; JN226765; AB605240; AB333801; HM770721; HQ883276; JF799911; JN162672; JN162671; JN935766; JQ326208; AP012285; AP012302.

### Genbank accession numbers of adenovirus sequences determined in this study

hu4108_DRC: KF976521; hu4109_DRC: KF976522; hu4214_DRC: KF976523; hu4152_DRC: KF976524; hu4719_IC: KF976525; hu4746_IC: KF976526; hu4751_IC: KF976527; hu4787_IC: KF976528; hu4806_IC: KF976529; hu4813_IC: KF976530; hu4882_IC: KF976531; hu4555_UG: KF976533; hu4557_UG: KF976532; hu4564_UG: KF976534.

## Competing interests

The authors declare that they have no competing interests.

## Authors’ contributions

MP, LM, KP, CAK, ECH, AEA, AM, GS, LW and PS collected the study samples and performed the questionnaires. FHL, BE, CAK, ECH, GS, JJM and SK have been involved in the conception of the study and coordinated the field work. MP, BE, EH, SW, SAL, SCS and FHL implemented the laboratory, statistical and phylogenetic analyses of the data and have contributed to the draft and revision of the manuscript. All authors have read and approved the final manuscript.

## Supplementary Material

Additional file 1: Table S1 Published studies on adenovirus prevalence in children with gastroenteritis. Click here for file
