# Supplementary material for: High prevalence and diversity of species D adenoviruses (HAdV-D) in human populations of four Sub-Saharan countries
Source: Virol J. 2014 Feb 11;11:25. doi: 10.1186/1743-422X-11-25 (PMC3928611; doi:10.1186/1743-422X-11-25)
Supplement: Additional file 1: Table S1 — Published studies on adenovirus prevalence in children with gastroenteritis. [file 1743-422X-11-25-S1.doc]

**Supplementary Table 1 Published studies on adenovirus prevalence in children with gastroenteritis**

| **Country** | **Setting** | **Hospitalized** | **Age (years)** | **Symptoms** | **Sample type** | **Samples tested** | **Test** | **HAdV-positive samples (%)** | **SequencedHAdV-positive samples** | **HAdV-D positive samples (%)** | **Ref.** |
| --- | --- | --- | --- | --- | --- | --- | --- | --- | --- | --- | --- |
| Bangladesh | urban | ? | children (0–3) | gastroenteritis | stool | 917 | PCR | 17 (2) | 17 | 10 (1.1) | [25] |
| Thailand | urban | yes | children (0–15) | gastroenteritis | stool | 1138 | PCR | 69 (6) | 76 | 2 (0.2) | [26] |
|  |  |  | adults (>15) |  |  | 124 |  | 2 (2.6) |  |  |  |
| Botswana | urban | ? | children (0–5) | gastroenteritis | stool | 346 | ELISA | 27 (8) | n.d | n.d. | [27] |
| Brazil | urban | yes | children (0–5) | gastroenteritis | stool | 3060 | EIA | 61 (2) (EIA) | 59 | 3 (0.09) | [28] |
| Burkina Faso | urban | yes | children (0–5) | gastroenteritis | stool | 66 | Microscopy and immunochromatography | 1 (1.5) | n.d | n.d. | [29] |
| France | urban | yes | Adults and children (?) | gastroenteritis | stool, urine, respiratory | 2300 | qPCR, PCR | 92 (4) | 57 (randomly chosen) | 1 (0.07) | [30] |
| Germany | urban | yes | children (?) | gastroenteritis | stool | 129 | PCR | 31 (24) | 31 | 0 (0) | [31] |
|  |  | no |  | none | stool | 28 |  | 3 (14) | 3 | 0 (0) |  |
| Kenya | urban | yes | children (0–14) | gastroenteritis | stool | 137 | PCR | 60 (43.8) | 55 | 18 (13.1) | [22] |
|  | rural |  |  |  |  | 80 |  | 34 (42.5) | 16 | 3 (3.8) |  |
|  | urban |  |  | none |  | 61 |  | 10 (16.4) | 7 | 1 (1.6) |  |
|  | rural |  |  |  |  | n.d. |  | n.d. |  | n.d. |  |
| Ghana | peri-urban | yes | children (0–11) | gastroenteritis | stool | 367 | PCR | 73 (19.9) | 73 | 23 (6.3) | [23] |
| Nigeria | urban | yes | children (0–5) | gastroenteritis | stool | 138 | Cell Culture and EIA | 23 (17) | n.d | n.d | [32] |
|  |  |  |  | none |  | 29 |  | 4 (14) |  |  |  |
| Tunisia | ? | yes | children (0–5) | gastroenteritis | stool | 638 | EIA and ELISA | 35 (6) | n.d | n.d | [33] |
| France | urban | yes | children (0–4) | gastroenteritis | stool | 973 | EIA | 49 (5) | n.d | n.d | [34] |
| Japan | urban | no | children (?) | gastroenteritis | stool | 88 | PCR | 11 (12.5) | n.d. | n.d. | [35] |
|  |  |  |  | none |  | 833 |  | 96 (41.7) |  |  |  |

**n.d.: not determined.**
